# Ranibizumab Port Delivery System in Neovascular Age-Related Macular Degeneration: Where Do We Stand? Overview of Pharmacokinetics, Clinical Results, and Future Directions

**DOI:** 10.3390/pharmaceutics16030314

**Published:** 2024-02-23

**Authors:** Matteo Mario Carlà, Maria Cristina Savastano, Francesco Boselli, Federico Giannuzzi, Stanislao Rizzo

**Affiliations:** 1 Ophthalmology Department, Fondazione Policlinico Universitario A. Gemelli, IRCCS, 00168 Rome, Italy; mariacristina.savastano@gmail.com (M.C.S.); francescoboselli@outlook.it (F.B.); federico.giannuzzi@gmail.com (F.G.); stanislao.rizzo@gmail.com (S.R.); 2 Ophthalmology Department, Catholic University “Sacro Cuore”, 00168 Rome, Italy

**Keywords:** anti-VEGF, port delivery system, ranibizumab, neovascular age-related macular degeneration, intraocular drug administration, intravitreal injection, ocular drug release systems, pharmacokinetics

## Abstract

The ranibizumab (RBZ) port delivery system (PDS) is a device designed to continuously deliver RBZ in the vitreous chamber for the treatment of neovascular age-related macular degeneration (nAMD). It is implanted during a surgical procedure and can provide sustained release of the medication for several months. This review, updated to January 2024, focuses on past clinical studies as well as current and forthcoming trials looking into a PDS with RBZ. In the phase 2 LADDER trial, the mean time to first refill of a PDS with RBZ 100 mg/mL was 15.8 months, with the pharmacokinetic (PK) profile showing a sustained concentration of RBZ in the blood and aqueous humor. More recently, a PDS with RBZ (100 mg/mL) refilled every 24 weeks was shown to be non-inferior to a monthly intravitreal injection (IVI) with RBZ (0.5 mg) over 40 and 92 weeks in the phase 3 ARCHWAY trial. The refill every 24 weeks allowed for a RBZ vitreous exposure within the concentration range of monthly intravitreal injections (IVIs), and the expected half-life (106 days) was comparable with the in vitro results. Nonetheless, vitreous hemorrhage and endophthalmitis were more common side effects in PDS patients. In conclusion, a PDS continuously delivering RBZ has a clinical effectiveness level comparable with IVI treatment. However, a greater frequency of unfavorable occurrences highlights the need for procedure optimization for a wider adoption. Ongoing trials and possible future approaches need to be addressed.

## 1. Introduction

In high-income nations, age-related macular degeneration (AMD) is the primary cause of blindness [1]. According to forecasts, 288 million people will be affected by AMD by 2040, placing a significant strain on healthcare systems [2].

Neovascular AMD (nAMD, also known as “wet AMD”) is the main cause of visual loss in contrast to “dry”, non-neovascular AMD. Due to their proangiogenic properties, growth factors generated by the retinal pigment epithelium (RPE) such as vascular endothelial growth factor (VEGF) are essential in influencing the pathophysiology of nAMD and thus have been targeted as therapeutic options [3]. Targeting VEGF-A, ranibizumab (RBZ) (Lucentis, Genentech, Inc., South San Francisco, CA, USA) is a fragment of a humanized monoclonal antibody that inactivates VEGF receptors 1 and 2, hampering the downstream signaling cascade of angiogenesis and inflammation [4]. The Food and Drug Administration (FDA) approved RBZ in 2006 for the treatment of nAMD, based on the key ANCHOR and MARINA trials [5,6].

In clinical trials, anti-VEGF-treated participants showed constant 1- to 2-line vision improvements from the baseline, with patients who received monthly treatments and monitoring showing the greatest effect [7,8,9]. On the other hand, visual improvements over the baseline are often restricted to less than one line of vision in real-world studies [10,11,12]. The substantial treatment burden connected to nAMD care and therapy may contribute to part of the discrepancy between the clinical trial findings and clinical practice outcomes [13,14]. Therefore, new strategies for sustained VEGF suppression have been assessed.

The port delivery system (PDS) with ranibizumab (Susvimo, Genentech, Inc., South San Francisco, CA, USA) is a cutting-edge, long-acting drug-delivery device that allows for the continuous release of a personalized formulation of RBZ into the vitreous, potentially reducing the treatment burden while preserving good visual results. This permanent, refillable implant is surgically placed via a tiny incision in the pars plana and sclera, and the replenishment of its reservoir is permitted by a self-sealing septum located in the middle of the implant flange. Through a porous metal release-control device, RBZ passively diffuses from the implant reservoir along a concentration gradient and into the vitreous cavity [15]. Following the phase 2 LADDER study [15] and the phase 3 ARCHWAY trial [16], the FDA, on 22 October 2021, authorized the PDS with RBZ for patients with nAMD who had responded to at least two anti-VEGF therapies. Following FDA approval, intriguing new clinical trials are being planned.

The aim of this review is to outline the situation regarding the art of PDS application in ophthalmology practice, focusing on clinical trial results, pharmacokinetics, adverse events, and future perspectives.

## 2. Methods

We examined the Cochrane Central, PubMed, Web of Science, and ClinicalTrials.gov databases for papers and guidelines regarding the ranibizumab PDS. Databases were accessed on January 2024. We only took into account English-language articles. None of the writers performed any new investigations involving humans or animals; instead, this narrative review was solely based on earlier research.

## 3. The Device

**Figure 1:**
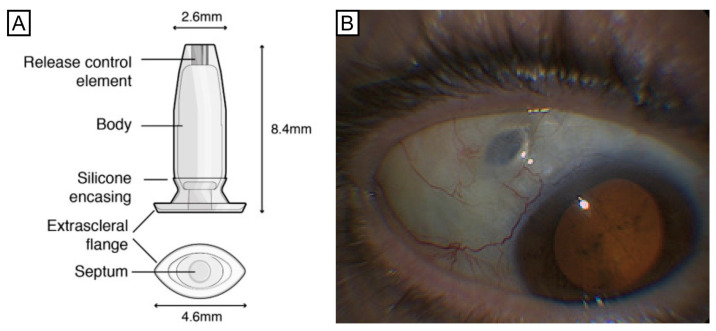
(**A**) Schematic figure of the ranibizumab port delivery system (PDS), detailing components and dimensions measured in millimeters. (**B**) Slit lamp photography of implanted PDS in the supero-temporal scleral quadrant.

The PDS is a hollow drug reservoir made of a non-biodegradable silicone-coated polysulfone body that can retain 0.02 mL of RBZ. An extrascleral flange anchors the PDS inside the sclera. This flange is a flaring of the silicone encasement and it is designed to sit superficial to the sclera and anchor the device deep into the conjunctiva without the need for sutures (Figure 1). The implant has dimensions of 8.4 mm in length, 4.6 mm at the flange, and 2.6 mm at the tip [17]. The implant is made of polysulfone and presents a self-sealing septum in the middle of the implant flange (proximal end) that maintains access to the implant reservoir (0.02 mL) for medication replenishment. At the distal end, a semipermeable titanium membrane enables the medicine to continuously diffuse into the vitreous. Fick’s law of diffusion is the physical principle whereby passive diffusion into the vitreous cavity occurs due to presence of a concentration gradient between the higher concentration of RBZ in the implant and the lower concentration in the vitreous. The medication contained in the PDS is refillable in a clinic using a special dual-bore needle inserted through the PDS septum that enables the simultaneous removal of any remaining RBZ from the reservoir and the injection of the new solution. The self-sealing septum is made of silicone [18,19].

## 4. Surgical Technique

As described in the LADDER trial, implant insertion must be performed in the operating room under local anesthetic. The original surgical technique involved conjunctival peritomy in the supero-temporal quadrant, followed by a stab incision on the pars plana 4 mm posterior to the limbus [15]. An optimized surgical technique was included in the May 2016 instructions for use procedure update. The minimum size of the peritomy should be 6 × 6 mm, with limbal and radial edges forming a 90° arc. The selected area for the implant insertion site should be closer to the peritomy flap base. The scleral dissection is followed by ablation of the exposed pars plana with a 532 nm laser (starting at 300 mW power and 1000 ms duration) in order to reduce post-implantation choroidal bleeding and vitreous hemorrhage [20]. Moreover, an additional diathermy can be performed as needed. The pars plana is then full-thickness-incised using a 3.2 mm slit knife to reach the vitreous cavity. After filling the implant in the operating room with RBZ, it is inserted in the scleral wound using the PDS insertion tool. Successively, the PDS must be carefully sutured to the conjunctiva and Tenon’s capsule to ensure that the implant flange is well covered [15].

Ericksen et al. recently reported that anterior segment optical coherence tomography (AS-OCT) can be used to follow eyes with the PDS implant in order to assess the correct position and orientation as well as possible scleral thinning [21].

### Refill–Exchange Technique

The PDS refill–exchange procedure is performed in aseptic conditions under topical anesthesia. To perform the procedure, clinicians require a 1 mL syringe and a special needle comprising a 34 G stainless-steel cannula with ventilated sleeves, a reservoir to collect the fluid, and a soft stop [18]. Ranibizumab is transferred from the vial in a sterile manner using a filter needle and any air in the syringe is eliminated. Subsequently, the filter needle is substituted with the refill needle, and any residual air is extracted from the syringe. The plunger is then pushed forward until it reaches the 0.1 mL indicator. To target the center of the implant septum, the refill needle is inserted perpendicular to the globe until it makes contact with the conjunctiva. The perpendicular orientation and contact with the conjunctiva are maintained throughout the procedure. It is recommended that a cotton-tipped applicator is used to stabilize the globe [18].

Through apertures in the vented needle, the existing content in the reservoir moves toward the fluid-collecting reservoir when fresh RBZ is administered. The amount of fresh medication injected into the reservoir (0.1 mL) must be five times larger than the reservoir capacity (0.02 mL) in order to guarantee a refill–exchange efficiency of more than 98% [15]. The whole contents of the syringe are gradually administered over a period of 5–10 s, allowing the current solution to fill the fluid-collecting reservoir. After finishing, the needle is cautiously removed while ensuring it remains perpendicular [18].

Timmons et al., when using AS-OCT, highlighted a case of implant septum dislodgement. At each refill, the images showed an increased distance between the septum from the overmold and, consequently, the refill procedures were stopped [22].

## 5. Pharmacokinetics

RBZ is a humanized monoclonal antibody fragment that binds to and blocks VEGF-A. Elevated levels of VEGF-A are found in the vitreous fluid of patients with wet age-related macular degeneration, diabetic retinopathy, macular edema, and neovascular glaucoma. VEGF is a signaling molecule that stimulates angiogenesis in the body and in the eye, and hypoxic cells are primarily responsible for the production of VEGF due to elevated interstitial pressure. Ranibizumab disrupts the binding of VEGF-A to its receptors, inhibiting the formation of new blood vessels [4].

After classic IVIs, the vitreous elimination half-life (t_1/2_) of RBZ is 9 days, whereas the intrinsic systemic elimination t_1/2_ is about 2 h. After IVI, RBZ slowly exits the eye and enters the bloodstream, leading to an observed serum half-life of 9 days. The systemic-to-vitreous exposure ratio was calculated to be 1:90,000. Monthly and quarterly treatment schedules of RBZ resulted in blood concentrations of the drug at a steady-state that were below the required level to suppress VEGF-A-induced endothelial cell proliferation by 50% for both the 0.3 and 0.5 mg/eye doses [23]. RBZ IVIs exhibit a phenomenon known as “flip-flop kinetics” [23]. In general, drugs are absorbed into the body faster than they are eliminated. However, flip-flop kinetics refers to a situation where the absorption rate is much slower than the rate at which the drug is eliminated from the body [24]. Conversely, results from in vitro research indicated that the PDS may provide a steady and consistent source of RBZ, which is determined by the initial concentration of RBZ. In a PDS arm loaded with 100 mg/mL, the release rate was 16.69 µg/day for 3.5 days and 4.16 µg/day for 6 months, indicating a stable and continuous release of the drug [25].

The phase 2 LADDER trial analyzed the pharmacokinetics (PKs) of the PDS in 68 patients (38% of the entire cohort). All patients had extensive blood sampling, which produced a solid dataset (5790 samples), allowing the ranibizumab PDS serum PK profile to be characterized. The results showed that, until at least month 9, the concentrations in the serum were within the expected range of monthly intravitreal RBZ 0.5 mg IVIs (130–2220 pg/mL) [7,8], staying above the expected serum trough concentration (C_trough_) and below the expected maximum concentration (C_max_) in the PDS 100 mg/mL group (235 pg/mL), while being below the C_trough_ in the PDS 10 mg/mL group (27.9 pg/mL) and near the expected C_trough_ in the PDS 40 mg/mL group (110 pg/mL) [15,26]. For all PDS arms (10, 40, and 100 mg/mL), there was an approximate correlation between the serum concentration and the aqueous humor concentration [26].

The mean highest concentration for patients receiving a 100 mg/mL PDS-based treatment at implantation was around 1080 pg/mL. Furthermore, in the PDS 100 mg/mL arm, the serum ranibizumab concentration remained above the lower limit of quantification (LLOQ) until month 16, not showing the aforementioned flip-flop kinetics [26]. Finally, a non-compartmental analysis of the LADDER trial’s serum data revealed a RBZ half-life of 88–168 days [26].

**Figure 2:**
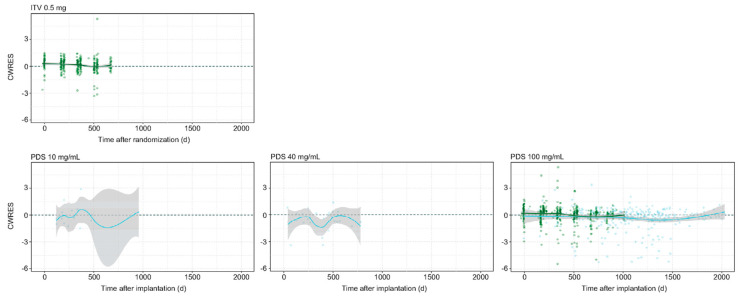
Graphs showing pharmacokinetics of classic ranibizumab (RBZ) 0.5 mg intravitreal injection (IVI) compared with the port delivery system (PDS) in the aqueous humor. (Courtesy of Kagedal et al.) [27]. Conditional weighted residual (CWRES) versus time after implantation (for PDS arms) or time after randomization (IVI arm) for aqueous humor nominal dose across clinical trials. Solid line indicates locally estimated scatter plot smoothing (LOESS), with the surrounding shaded area representing the 95% confidence interval. The blue points are the patients initially enrolled in the LADDER trial; the green ones are patients initially enrolled in the ARCHWAY trial.

More recently, Kagedal et al. developed a population PK model, gathering data from the LADDER, ARCHWAY, and ongoing PORTAL trials and using a non-linear mixed-effect modeling approach (NONMEM) [27]. The dataset used for this population PK study consisted of samples from a total of 164 patients from LADDER, 276 patients from ARCHWAY, and 110 patients from PORTAL (54 of whom were recruited from LADDER and 56 from ARCHWAY), with a total of 4069, 2016, and 488 serum concentration observations acquired from the three trial groups, respectively. As a result, the predicted half-life of RBZ release from the PDS was 106 days, which was comparable with the half-life found from the in vitro data (99 days for a PDS of 100 mg/mL) [25]. According to the model, when using refill–exchange procedures every 24 weeks (Q24W), the vitreous exposure to RBZ fell within the minimum and maximum (C_min_ and C_max_) concentration range of monthly intravitreal 0.5 mg RBZ IVIs [28,29]. Regarding aqueous humor concentrations, the observed and expected concentration release rates were comparable, with the estimated aqueous-to-vitreous concentration ratio being similar to that reported for other antibody fragments such as lampalizumab [30] (Figure 2).

In addition, as the observed serum half-life appeared to be longer than the in vitro data, Kagedal et al. included a time-varying clearance from the serum that decreased with time, hypothesizing that the development of acidic RBZ variants in the PDS implant would have a lower clearance (CL) than the original one in the refill solution. These acidic variants, already demonstrated in in vitro investigations, do not impact on vitreous clearance [31]. On the other hand, as RBZ is small enough to be filtered by the kidney, it is conceivable that the serum clearance of ranibizumab might be sensitive to charges via electrostatic interactions in the kidney [23]. Moreover, as each refill–exchange procedure washes out any RBZ variants from the implant, the clearance reverts to original values; thus, they only vary within dosage intervals [27]. Finally, the estimated vitreous elimination half-life of 5.8 days fell between the previously reported values of 5.8 and 8.6 days [23,32,33].

### Biocompatibility

Bantseev et al. conducted an extensive battery of in vitro and in vivo biocompatibility testing on the PDS, revealing that the implant exhibited no cytotoxicity, gene toxicity, sensitization, or irritation. The implant did not cause any localized inflammation or other reactions when it was inserted into rabbit muscle tissues. Ancillary device extracts were shown to be non-cytotoxic, non-sensitizing, and non-irritating; as a result, they were regarded as biocompatible. Furthermore, there were no additional safety concerns about toxicity associated with the administration of ranibizumab through a PDS implant rather than classic intravitreal injections (IVIs) [34].

## 6. Clinical Results

### 6.1. The LADDER Trial

The randomized, multicenter, active-treatment controlled phase 2 LADDER study (NCT02510794) was initiated in 2015, supported by Genentech, Inc. [15,35,36]. The trial’s objective was to assess the PDS’s safety and effectiveness in 220 individuals with nAMD. Patients were randomized in a 3:3:3:2 fashion to receive monthly IVIs with 0.5 mg/mL RBZ or a PDS with RBZ 10 mg/mL, 40 mg/mL, or 100 mg/mL [15].

In the monthly assessments, a rise in the central foveal thickness (CFT), a fall in best-corrected visual acuity (BCVA), or a macular hemorrhage were indications for an implant refill. Consequently, the primary endpoint was the time to first refill. For the PDS arms with 10 mg/mL (*n* = 58), 40 mg/mL (*n* = 62), and 100 mg/mL (*n* = 59), the median time to the first refill was 8.7, 13.0, and 15.8 months, respectively. After a year, the percentages of patients who did not obtain a refill at 10, 40, or 100 mg/mL were 28.9, 56.0, and 59.4%, respectively [15].

Changes in the CFT and BCVA were included in the secondary outcomes. After 22 months of follow-up, the mean decrease in BCVA from the baseline, measured using early treatment diabetic retinopathy study chart (ETDRS) letters, was −4.6 letters, −2.3 letters, +2.9 letters, and +2.7 ETDRS for PDSs of 10, 40, and 100 mg/mL and monthly IVIs, respectively. Additionally, at month 22, the baseline visual acuity was maintained by 57.7%, 80.0%, 87.5%, and 88.9% of patients receiving PDSs of 10, 40, and 100 mg/mL and monthly IVIs, respectively. At month 22, patients had CFT values of −0.7 µm, −20.9 µm, −4.0 µm, and −10.9 µm in the 10, 40, 100 mg/mL PDS groups and those receiving monthly IVIs, respectively [15].

Moreover, an exploratory analysis of the phase 2 LADDER trial analyzed the prevalence of macular atrophy (MA) across the treatment groups, showing comparable results for the 10, 40, and 100 mg/mL PDS arms [37]. The prevalence of MA at 22 months was comparable between the monthly RBZ group (45.7%) and PDS arms (38.6%, 40.0%, and 40.4% for 10, 40, and 100 mg/mL PDSs, respectively), with no significant difference in the size of MA lesions between the PDS groups and monthly IVI group at each follow-up. Although a large variability was reported, the PDS 10 mg/mL arm showed the most improvement in the area of MA from the baseline [37].

Moreover, in patients without MA at the baseline, a higher proportion of eyes in the monthly RBZ arm (40.6%) developed MA compared with the 100 mg/mL PDS group (30.6%), suggesting that the continuous administration of MA may be linked to lower rates and slower development of MA compared with bolus intravitreal injections. However, the trial lacked the capacity to identify significant differences in the incidence or development of MA across the treatment groups, and the rate of MA was a prospectively designed exploratory outcome [37].

### 6.2. The ARCHWAY Trial

The open-label, randomized, non-inferiority phase 3 ARCHWAY study (NCT03677934), funded by Genentech, Inc. [16,38], recruited a total of 418 patients, assigned in a 3:2 randomization to receive monthly RBZ IVIs (*n* = 167) or a 100 mg/mL PDS Q24W (*n* = 251, of which *n* = 248 were treated). To be included, patients needed previous anatomical and visual responses to anti-VEGFs (RBZ, bevacizumab, or aflibercept) within the preceding 6 months.

The primary goal of the study was to determine the non-inferiority (±4.5 ETDRS letters) of the 100 mg/mL PDS in terms of BCVA changes (in ETDRS) at weeks 36 and 40. The adjusted mean changes from the baseline were +0.2 letters for the PDS and +0.5 letters for the monthly IVI groups, respectively (mean difference of −0.3 letters) [16]. The visual and anatomical results of BCVA and center point thickness (CPT) were included in the secondary endpoints. The changes in CPT with the PDS Q24W (5.4 µm) were comparable with those of monthly RBZ (2.9 µm) at week 36 (difference of 2.8 µm). Up to week 40, the two treatment groups’ CPT levels were statistically equivalent. Finally, in the PDS group, 98.4% (*n* = 246) of the patients did not need further IV treatment prior to the first refill at 24 weeks [16].

An expansion of ARCHWAY over 2 years, supported by Genentech, Inc., included 248 individuals in the 100 mg/mL PDS group and 167 in the IVI-based group [39]. Overall, the findings agreed with ARCHWAY’s main analysis. According to reports, 13 patients receiving IVI-based therapy and 24 patients receiving PDS-based therapy stopped their treatments. Termination was due to adverse events, withdrawal, and death.

The same main goal of ARCHWAY was pursued. The mean changes in BCVA over weeks 60 and 64 as well as—upon request from the European Medicines Agency—during weeks 44–48 and 88–92 were secondary objectives. The adjusted mean changes for the PDS-treated patients were 0.0 letters, −0.4 letters, and −1.1 letters from the baseline to weeks 44 to 48, 60 to 64, and 88 to 92, respectively. At the same time points, the changes in BCVA for patients receiving monthly RBZ were 0.2 letters, −0.8 letters, and −0.5 letters. As a consequence, considering a non-inferiority margin of ±3.9 ETDRS letters, the PDS-based therapy with RBZ (100 mg/mL) demonstrated non-inferiority to the monthly IVI-based treatment with RBZ (0.5 mg) [39]. In the PDS group, during the first 24 weeks, 98.4% of patients did not need further IVI treatment [26] and 94.7% of patients did not require additional IVI therapy during the final 24 weeks (weeks 73–96). Up to week 96, the CPT difference in the PDS group was 11.3 µm and there was no statistically significant difference between the treatment groups [39].

An overview of the LADDER and ARCHWAY trial results is presented in Table 1.

## 7. Adverse Events

Overall, according to LADDER, patients undergoing the PDS-based treatment had more adverse events (AEs) than those undergoing IVI-based therapy. In the first 37 days after implantation, 89.9% of patients treated with the PDS and 9.8% of patients treated with IVI had adverse events [15]. Conjunctival hemorrhage was the most common non-serious adverse event across all arms at study completion, accounting for 70.69, 70.97, 62.71, and 19.51% of patients receiving 10, 40, and 100 mg/mL PDSs and IV with RBZ 0.5 mg, respectively. Upon the study’s conclusion, 9.5% of patients undergoing PDS-based therapy and none of the patients receiving monthly IVI-based therapy had encountered several adverse events (SAEs). Although a definition of an SAE was not provided by LADDER, vitreous hemorrhage was the most often reported SAE. Prior to May 2016, 11/22 (50%) of the previously evaluated patients receiving a PDS-based treatment had a significant incidence of vitreous hemorrhage, which caused the trial suspension. After surgical optimization in May 2016, the rate of vitreous hemorrhage in the PDS arm decreased to 8/157 (5.1%). Moreover, compared with none of the patients receiving IVI-based treatment, three patients undergoing the PDS treatment developed endophthalmitis [15].

In the ARCHWAY trial, similarly, the incidence of AE and SAE was greater in patients receiving the PDS-based treatment (94.4% for AE and 5.6% for SAE) than in patients receiving IVI-based therapy (35.5% for AE and 1.2% for SAE) at week 40. SAEs were defined as a death, a danger to life, an extended hospital stay, a congenital birth defect in one study participant’s infant, a permanent or substantial handicap, or any other occurrence determined by an investigator. Thirteen (5.2%) patients receiving the PDS-based treatment experienced vitreous hemorrhage, predominantly occurring in the first month, compared with four (2.4%) in the IVI arm. Four PDS patients (1.6%) experienced endophthalmitis, all occurring outside the first month, compared with no cases in the IVI group [16]. In the extension up to week 96, 4 patients receiving IVI-based therapy suffered an SAE (2.4%) compared with 22 patients receiving the PDS-based therapy (8.9%). Moreover, 15 individuals of the PDS arm suffered vitreous hemorrhage and 4 developed endophthalmitis. In the IVI arm, six patients experienced vitreous hemorrhage and one patient developed endophthalmitis [39].

A post hoc analysis of PDS-related AEs was performed by Awh et al. and Pieramici et al., who proposed an optimized step-by step surgical procedure. For example, a reduction in the vitreous hemorrhage rates was achieved by adding pars plana laser ablation before a pars plana incision. Although pars plana laser ablation may lower the incidence of vitreous hemorrhage, hemostasis must be carefully monitored and controlled at every stage. Thus, it is important to take precautions during the laser ablation process to ensure the laser does not widen the incision, particularly when lasering the sclera in the corners. Nonetheless, because the corner may be a source of bleeding, the cautious laser ablation of the pars plana in the corners is crucial. This was a step forward compared with the originally unacceptable rate of vitreous hemorrhage linked with the implant insertion process in the LADDER study [40]. Comprehensive video and image assessments of each PDS implant insertion and refill–exchange process have also proven to be a useful tool in the PDS clinical trials in order to optimize the techniques over time. For example, a scleral incision longer than the advised 3.5 mm might raise the risk of implant displacement during the refill–exchange operation. Tissue handling, implant positioning away from the radial incision, and closure procedures were given more attention in order to lower the risk of tissue damage and conjunctival erosion and retraction, which could also lower the risk of post-operative endophthalmitis [20]. Moreover, a non-complete suture of Tenon’s capsule and conjunctiva could increase the risk of later conjunctival erosion or retraction. Erosion and retraction risk may be decreased by completely closing the conjunctiva and Tenon’s capsule using scleral anchoring and leaving a small amount of tissue overhanging (about 1–2 mm) the anterior limbus. A little overhang of tissue prevents the natural retraction that is anticipated throughout the healing process and permits implant flange coverage [20].

Finally, during the refill–exchange procedure, twisting the syringe could increase the risk of injury to the implant and surrounding tissue [18].

Therefore, updated guidelines and suggestions have been supplied for the PDS implant insertion process, refill and exchange method, appropriate conjunctiva care, and Tenon’s capsule handling practices. Additionally, physicians undergoing PDS surgical training have access to cutting-edge virtual reality teaching technology that was specifically developed for the PDS [40,41].

## 8. Future Directions

An overview of the ongoing clinical trials regarding the PDS is available in Table 2. All trial data were accessed on 18 January 2024.

The PAGODA trial (NCT04108156) is a multicenter, randomized, phase 3 clinical trial that examines the safety, pharmacokinetics, and effectiveness of the PDS in patients with diabetic macular edema (DME) when treated every 24 weeks (Q24W) compared with IVI 0.5 mg RBZ every 4 weeks (Q4W). The main outcome is the change in BCVA from the baseline to weeks 60 and 64. Launched in 2019, PAGODA is expected to conclude in 2024 [42].

The PAVILION trial (NCT04503551) is a randomized, multicenter, phase 3 clinical trial studying the safety, efficacy, and pharmacokinetics of the 100 mg/mL PDS refilled every 36 weeks (Q36W) in patients with diabetic retinopathy (DR) without center-involved diabetic macular edema (CI-DME) compared with IVI 0.5 mg RBZ. The primary endpoint is the rate of patients with at least a 2-step improvement in the ETDRS chart–Diabetic Retinopathy Severity Scale (ETDRS-DRSS) 52 weeks from the baseline. The PAVILION study started in 2020 and is expected to conclude in 2024 [43].

The VELODROME trial (NCT04657289) is an active-comparison, phase 3b, multicenter, randomized investigation to assess the pharmacokinetics, safety, and effectiveness of the PDS with RBZ (100 mg/mL) refilled every Q36W versus Q24W in nAMD patients. The change in ETDRS BCVA from the baseline, averaged across weeks 68 and 72, is the main outcome. BCVA is evaluated using the ETDRS chart. The VELODROME study is expected to conclude in 2026, having begun in 2021 [44].

The open-label, multicenter PORTAL trial (NCT03683251) by Hoffmann La-Roche aims to examine the long-term safety and tolerability of the PDS with RBZ (100 mg/mL) in patients with nAMD who have participated in the LADDER, ARCHWAY or VELODROME studies. Using data up to week 240, the major outcomes examine the frequency, intensity, and duration of ocular and non-ocular adverse events (AEs). The patients will receive a Q24W PDS, except for patients from VELODROME who will be assigned to Q36W and who will keep this interval. The PORTAL trial began in 2018 and is expected to conclude in 2026 [45].

The phase 4, multicenter, open-label BELVEDERE study (NCT04853251) will assess changes in corneal endothelial cells in nAMD treated with a 100 mg/mL PDS and Q24W refills. The study started in 2021 and its estimated completion date is 2027 [46].

The VOYAGER trial (NCT05476926) is a prospective, non-interventional, multinational study gathering long-term real-world data from patients treated for five years with Roche ophthalmology products (faricimab or the PDS with RBZ), with the goal to evaluate the clinical insights, safety, and effectiveness of the two. The main endpoint is a change in BCVA, utilizing the local technique of BCVA acquisition up to a year, after which BCVA is translated into ETDRS letters. The VOYAGER investigation is scheduled to conclude in 2027, having begun in 2022 [47].

The DIAGRID trial (NCT05126966) was a multicenter, randomized, phase 3b research study, with the objective of comparing the safety and effectiveness of the PDS with RBZ (100 mg/mL), refilled Q36W, with an IVI-based treatment with aflibercept (2 mg) in a T&E (treat-and-extend) regimen in patients with nAMD. The main outcomes were the frequency of therapy up to week 80 and the difference in BCVA between the baseline and week 80. As the implants from the commercial supply did not match the submitted requirements for intended usage in the clinical research, Roche/Genentech halted additional implantations. This has resulted in the suspension of the study [48].

Finally, a programmed randomized, multicenter, phase 3 clinical study trial on Chinese patients with nAMD (NCT05562947) will compare a monthly RBZ 0.5 mg IVI-based treatment with a PDS with RBZ (100 mg/mL), refilled Q24W. Similar to ARCHWAY, a change in BCVA from the baseline averaged across weeks 36 and 40 is the main outcome. Recruitment will start in 2024 and last until 2029 [49].

## 9. Conclusions

In conclusion, the PDS is a long-term medication reservoir that is implanted into the sclera to enable RBZ to be continuously delivered, perhaps lessening the burden of IVI therapy. With a mean time to first refill of 15.8 months, the 100 mg/mL PDS may constantly provide a level of RBZ falling between the C_min_ and C_max_ ranges of monthly IVIs. Moreover, the predicted half-life of RBZ release from the PDS (106 days) is consistent with in vitro data.

Nevertheless, the PDS has drawbacks such as higher rates of vitreous hemorrhage and endophthalmitis. Larger future research studies investigating the long-term effectiveness, safety, patient preference, and cost in real-world settings are required to fully comprehend the applicability of the PDS in daily practice.

## Figures and Tables

**Table 1:** Overview of the findings from the LADDER and the ARCHWAY trials.

	Phase 2 LADDER Trial	Phase 3 ARCHWAY Trial
Start/end	2015/2019	2018/2021
Participants	Overall, *n* = 220 PDS, *n* = 179 PDS with RBZ (10 mg/mL, *n* = 58) PDS with RBZ (40 mg/mL, *n* = 62) PDS with RBZ (100 mg/mL, *n* = 59) IVI with RBZ (0.5 mg, *n* = 41)	418 randomized and 415 treated PDS, *n* = 248 IVI, *n* = 167
Intervention	PDS with RBZ (10, 40, or 100 mg/mL)	PDS with RBZ (100 mg/mL) refilled Q24W
Control group	Monthly IVI of 0.5 mg RBZ	Monthly IVIs of 0.5 mg RBZ
Follow-up period	22 months	36–40 weeks, then extended to 88–92 weeks
Primary findings	Median time to first refill PDS 10 mg/mL: 8.7 months (80% CI 6.9–9.0) PDS 40 mg/mL: 13.0 months (80% CI 11.8–24.6) PDS 100 mg/mL: 15.8 months (80% CI 12.1–20.6)	Mean change in ETDRS from baseline PDS arm: +0.2 letters (SE, 0.5) IVI arm: +0.5 letters (SE, 0.6) Difference: −0.3 ETDRS letters (CI, 1.7 to 1.1)
Secondary findings		
BCVA	Mean change in ETDRS from baseline PDS 10 mg/mL: −4.6 letters PDS 40 mg/mL: −2.3 letters PDS 100 mg/mL: +2.9 letters IVI 0.5 mg RBZ: +2.7 letters	Mean change in ETDRS from baseline to weeks 44 and 48 PDS Q24W: 0.0 letters (SE, 0.5) IVI: +0.2 letters (SE, 0.6) Mean change in ETDRS from baseline to weeks 88 and 92 PDS Q24W: −1.1 letters (SE, 0.6) IVI: −0.5 (SE, 0.8 letters)
OCT	Mean change in CFT from baseline PDS 10 mg/mL: −0.7 µm PDS 40 mg/mL: −20.9 µm PDS 100 mg/mL: −4.0 µm IVI 0.5 mg RBZ: −10.9 µm	Mean change in CFT from baseline at week 96 PDS Q24W: +9.9 µm (SE, 3.6) IVI: −1.3 µm (SE, 4.5)

PDS: port delivery system; RBZ: ranibizumab; BCVA: best-corrected visual acuity; OCT: optical coherence tomography; IVI: intravitreal injection; Q24W: fixed interval of 24 weeks; SE: standard error; CI: confidence interval; ETDRS: early treatment diabetic retinopathy study chart.

**Table 2:** Summary of ongoing and programmed clinical trials.

Trial	Start/End	Sponsor	Current Status	Condition	Participants	Study Type
PAGODA	2019/2024	Hoffmann La-Roche ^a^	Recruitment ended	DME	634 (actual)	Interventional
PAVILION	2020/2024	Hoffmann La-Roche ^a^	Recruitment ended	DME	174 (actual)	Interventional
VELODROME	2021/2026	Hoffmann La-Roche ^a^	Recruiting	nAMD	442 (estimate)	Interventional
PORTAL	2018/2026	Hoffmann La-Roche ^a^	Recruiting	nAMD	1000 (estimate)	Interventional
BELVEDERE	2021/2027	Genentech, Inc.	Recruiting	nAMD	185 (estimate)	Interventional
VOYAGER	2022/2027	Hoffmann La-Roche ^a^	Recruiting	nAMD	5000 (estimate)	Observational
DIAGRID	2023/2026	Hoffmann La-Roche ^a^	Suspended	nAMD	0 (actual)	Interventional
Chinese	2024/2029	Hoffmann La-Roche ^a^	Not yet recruiting	nAMD	68 (estimate)	Interventional

^a^ Basel, Switzerland. DME: diabetic macular edema; nAMD: neovascular age-related macular degeneration.

## Data Availability

Requests on data supporting this research should be addressed to the corresponding author, MMC.
